# Role of prefrontal cortex during Sudoku task: fNIRS study

**DOI:** 10.1515/tnsci-2020-0147

**Published:** 2020-11-03

**Authors:** Patil Ashlesh, Kishore K. Deepak, Kochhar Kanwal Preet

**Affiliations:** Department of Physiology, All India Institute of Medical Sciences, Nagpur, India.; Department of Physiology, All India Institute of Medical Sciences, New Delhi, India

**Keywords:** prefrontal cortex, Sudoku, problem solving, fNIRS, cognition, general linear model

## Abstract

**Background:**

Sudoku is a popular cognitively stimulating leisure-time activity. Many studies have been directed toward finding an algorithm to solve Sudoku, but the investigation of the neural substrates involved in Sudoku has been challenging.

**Methods:**

Sudoku task was divided into two steps to understand the differential function of the prefrontal cortex (PFC) while applying heuristic rules. PFC activity was recorded at 16 optode locations using functional near infrared spectroscopy. Classical two-way analysis of variance as well as general linear model-based approach was used to analyze the data from 28 noise-free recordings obtained from right-handed participants.

**Results:**

*Post hoc* analysis showed a significant increase in oxyhemoglobin concentrations and decrease in deoxyhemoglobin concentrations at all 16 optode locations during step 1 (3 × 3 subgrids) and step 2 (easy level 9 × 9 Sudoku) when compared with the rest (*p* < 0.0001). Contrasting the step 2 – step 1 revealed that medial regions of PFC were preferentially activated.

**Conclusion:**

Both the medial and lateral regions of PFC are activated during Sudoku task. However, the medial regions of PFC play a differential role, especially when we consider searching and selecting the heuristic rules. Thus, Sudoku may be used for cognitive remediation training in neuropsychiatric disorders involving PFC.

## Graphical abstract

**Figure:**
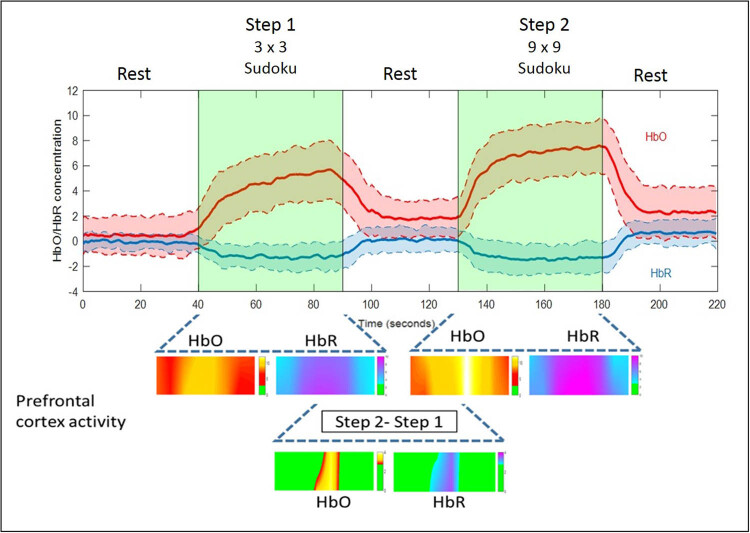

## 1 Introduction

One of the most common leisure-time activities for all ages is solving puzzles. Popular among these puzzles is Sudoku, a logic-based combinatorial number placement problem composed of a matrix with rows (*n* ^2^), columns (*n* ^2^), and subgrids (*n* × *n*) [1]. The problem is based on three simple rules that the numbers should not repeat in the subgrid, row, or column. Sudoku is a good cognitively stimulating leisure-time activity [2]. Sudoku requires attention of the subject to analyze the grids and fill in the numbers; basically it requires no math but is based on logic [1]. Solving puzzles has long been thought to keep the brain healthy [2] and has been shown to delay the onset of dementia [3]. Cognitive deficits are important features among many neuropsychiatric disorders such as depression, bipolar disease, schizophrenia, Parkinson’s and Alzheimer’s disease with dysfunctions in working memory, attention, reasoning, decision-making, and problem-solving [4,5,6,7]. Since solving Sudoku involves executive cognitive functions, most importantly problem-solving and decision-making, it can be a promising tool for neurorehabilitation and cognitive remediation therapy in neuropsychiatric disorders. However, little is known about the neural substrates involved during Sudoku.

Many studies have been directed toward finding an algorithm to solve Sudoku, but the investigation of the neural substrates involved in Sudoku has been challenging. Simplified versions of Sudoku (4 × 4 matrix) have been used in various functional magnetic resonance imaging (fMRI) studies [8,9,10,11,12]. Qin et al. found the involvement of the left prefrontal cortex (PFC) and dorsal anterior cingulate cortex and also the bilateral involvement of posterior parietal cortex, caudate nuclei, fusiform, and frontal eye field areas while solving the simplified version of Sudoku puzzle [9]. In fact, the same group of researchers had previously shown that the fMRI BOLD signal obtained from these regions of interest can be used to predict the mental states during puzzle solving [11]. Their unique Sudoku paradigm (4 × 4 matrix, 2 × 2 subgrid) eliminated the need of searching for the missing number or selecting the rules to be used. Rather, their paradigm was dedicated toward understanding the neural bases of the basic heuristic problem-solving processes in the knowledge domain. However, a complete Sudoku task includes both basic heuristic problem-solving processes and searching and selecting the heuristic rules. A 9 × 9 Sudoku requires the subject to be in a sitting posture for some time, which limits the use of traditional imaging techniques to study the brain activity during the task. In fMRI, the subject has to be motionless and in a cage of magnets, and this hardware restriction forms the ultimate limitation and restraints its use in claustrophobic participants. In addition, the supine motionless position is not the same used by an individual to solve the puzzle. The solution for such technical limitations is a perfectly noninvasive optical imaging technique called functional near infrared spectroscopy (fNIRS). The fNIRS is a simple, safe, and noninvasive neuroimaging technique of measuring brain activity [13]. The principle behind the technique is to measure the absorbance of the infrared light to calculate the relative ratios of deoxygenated and oxygenated hemoglobin by modified Beer–Lambert law [14]. This hemodynamic response provides the indirect information about the brain activity, as the neural activation and vascular response are tightly coupled, which is known as neurovascular coupling [15]. Another advantage of fNIRS over other imaging studies is its portability and robustness, which make it easy to study tasks akin to the daily routine activities [16]. The fNIRS fits the best technique to study puzzles such as Sudoku.

PFC is the most important area in the brain that participates in executive functions. Various functional neuroimaging studies have shown that PFC is involved in attention, working memory, decision-making, and problem solving [17]. Dysfunctions in PFC dynamics are observed in the neuropsychiatric disorders that lead to cognitive deficits [18]. fNIRS when applied to hair-free region of the forehead can record PFC activity during cognitive task. fNIRS has been shown to be a reliable tool to measure cognitive workload during working memory task [19]. Recent study has shown that fNIRS can be effectively used to study PFC dynamics during complex chess-based problem-solving tasks [20]. Since solving Sudoku also involves similar complex executive functions, we hypothesize that PFC is active during Sudoku when compared with the rest. Sudoku was uniquely divided into two steps to understand the differential role of the PFC while applying the heuristic rules.

## 2 Materials and methods

### 2.1 Participants

The participants with any known history of mental illness or neurodegenerative disorders such as dementia or any history of brain trauma or surgery were not included in the study. Thus, 33 apparently healthy individuals (9 females, 28.88 ± 2.50 years) with normal or corrected to normal vision and with previous exposure to Sudoku task were included in the study. Handedness was assessed by Edinburgh handedness inventory (31 participants were right-handed) [21].


**Ethical approval:** This study was approved by institutional ethical committee and in accordance with the Code of Ethics of the World Medical Association (Declaration of Helsinki 2000).
**Informed consent:** All the participants were well informed about the nature and purpose of the study, and written informed consent was obtained from each participant.

### 2.2 fNIRS imaging

**Figure 1:**
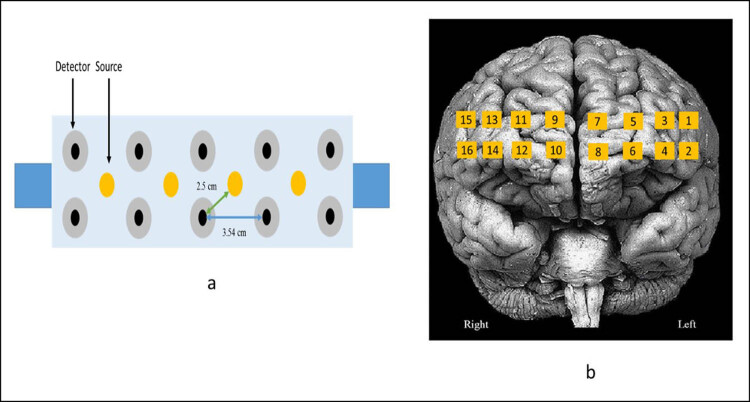
(a) fNIRS sensor pad: the sensor pad was placed on the forehead of the participants and aligned using 10–20 electrode placement system as mentioned in the text. The sensor pad consists of 4 sources and 10 detectors, which are configured to generate 16 optodes. (b) Optode locations: 16 optode locations are depicted on brain surface (frontal view).

Participants were seated comfortably in an armchair throughout the task while their prefrontal cortical activity was monitored using continuous-wave fNIR imaging system (Model 1100 BIOPAC Systems Inc.). The forehead was first cleaned by spirited cotton before placing the sensor pad. This pad consisted of 4 sources and 10 detectors, which are configured to generate 16 measurement locations on the forehead called optodes (Figure 1a). Fp1, Fp2, and Fz positions were marked using international 10–20 electrode placement system. The centerline of fNIRS sensor pad was first aligned according to Fz site with the horizontal axis coinciding with symmetry of the eyes and later it was adjusted in vertical plane, so that the lower optodes corresponded to Fp1 and Fp2 locations. Such a position registers the 16 optode locations, as depicted in Figure 1b [22]. The fNIRS sensor pad was stabilized with an elastic band to prevent displacement. The source in the sensor pad has LED lights with peak wavelengths at 730 and 850 nm, i.e., one above and other below the isosbestic point (∼800 nm) where absorption spectrums of oxygenated and deoxygenated hemoglobin cross. The contact of each source and detector was ensured by observing the record online. Gain and power of LED current were accordingly adjusted to obtain optimal intensities for all the wavelengths. After stabilization of the record, 10-s prescan baseline was recorded before the start of the task. Online data acquisition and visualization were done using COBI Studio software.

### 2.3 Study protocol

**Figure 2:**
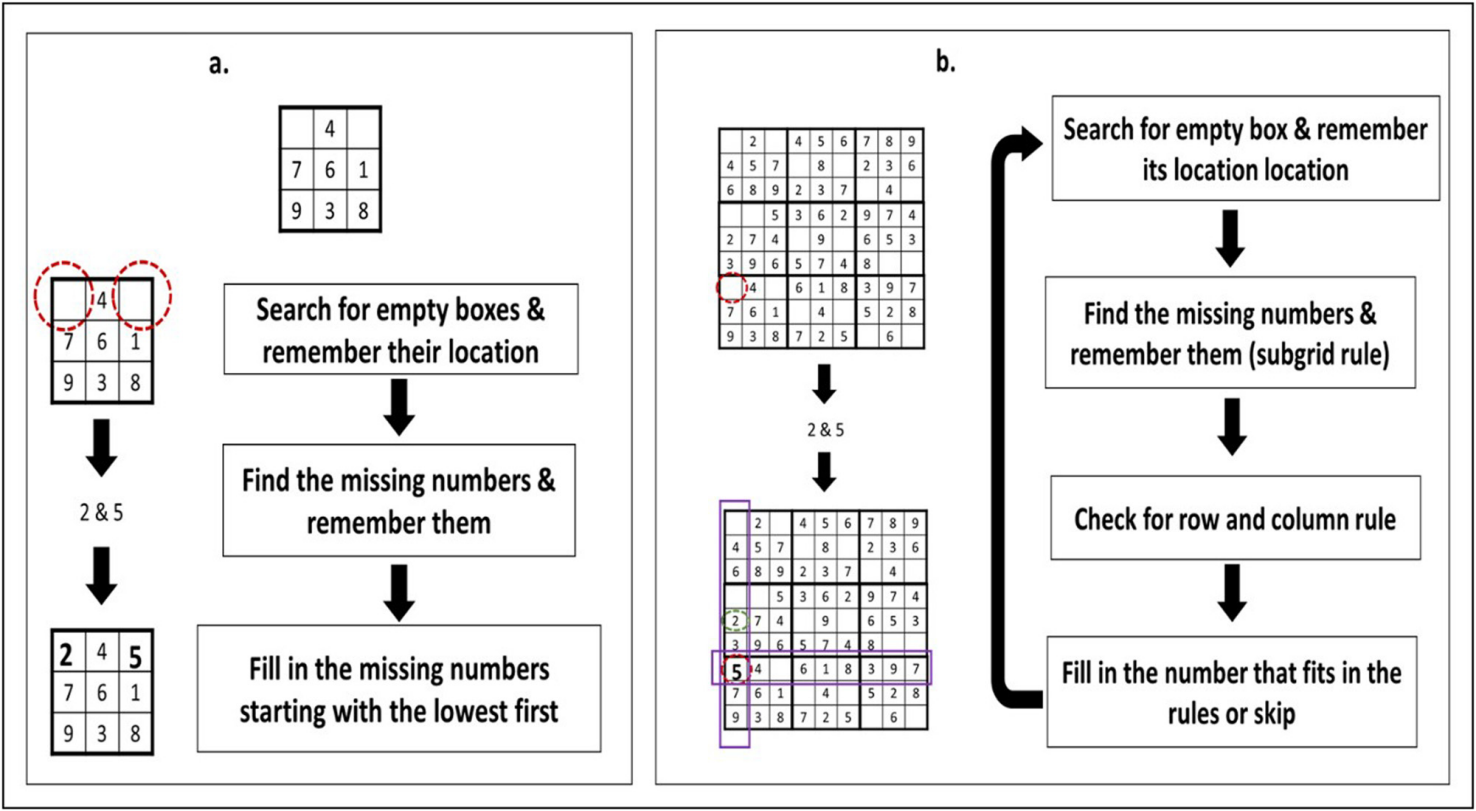
(a) Step 1, i.e., 3 × 3 subgrid: in this example, the participants need to fill in the missing numbers (2 and 5); (b) step 2, i.e., 9 × 9 subgrid: in addition, the participants need to fill in missing number according to the row and column rules.

After familiarization with the lab setup and practice sessions, participants were asked to perform the task using paper–pen technique (akin to daily routine) with continuous recording of the prefrontal activity. Sudoku task was divided into two steps (Figure 2). During step 1, the participant had to fill one, two, or three missing numbers (1–9) in the 3 × 3 grids starting from the first row, i.e., only the subgrid rule to be used. The participants had to complete 18 such grids (presented on the same paper sheet) with 33 total missing numbers for a period of 50 s. Step 2 consisted of an easy-level Sudoku (9 × 9 matrix with subgrids of 3 × 3) composed of 3 × 3 grids used in step 1. During step 2 apart from filling in the missing numbers, the participant also has to keep in mind the row and column rules of the Sudoku puzzle. After stabilizing the fNIRS recording, the task began with rest 1/baseline (40 s) → step 1 (50 s) → rest 2 (40 s) → step 2 (50 s) → rest 3 (40 s). These timings were decided from the results of the pilot study. Digital timer beeped at the end of each break and the participant was instructed to change the sheet. Blank sheets were used during the rest periods.

### 2.4 Data analysis and statistics

After data acquisition, analysis was done off-line using fNIRsoft software. Concentrations of oxyhemoglobin (HbO) and deoxyhemoglobin (HbR) were calculated using modified Beer–Lambert law with respect to the prescan baseline of 10 s. fNIRS data get easily corrupted by measurement noise and other physiological signals; and hence, careful statistical analyses are required to extract the neuronal-related activity from the signal [23]. Since statistical techniques for fNIRS are in nascent stage when compared with fMRI, classical methods need to be supplemented. In this study, we have used classical two-way analysis of variance (ANOVA) as well as general linear model (GLM)-based approach to give convergent evidence of PFC activity during Sudoku task. The data were first subjected to low-pass filter (0.40 Hz) with an order of 20 to remove trends due to respiratory, cardiovascular, or other high-frequency artifacts. Two-way ANOVA (3 task factors × 16 optodes) was used to compare the average concentration of HbO and HbR across the 16 optodes during baseline, step 1, and step 2. *Post hoc* analysis was done using Tukey’s multiple comparison test with an *α* value of 0.05. Statistical analysis was also done using mass-univariate approach based on the GLM in NIRS-SPM [24]. The task design was used to create a square wave with task periods as 1, which was then convoluted with hemodynamic response wave function to generate theoretical response. This theoretical response was compared with the actual response during statistical analysis. Wavelet minimum description length detrending algorithm was applied to the data to remove trends due to respiratory, cardiovascular, or other experimental errors. This method prevents over-/underfitting and facilitates optimal model order selection for the global trend estimate [25]. Precoloring method with low-pass hemodynamic response function filter was used for corrections in temporal autocorrelation [24]. Three contrast model matrices were assessed viz. contrast 1 (activity during step 1), contrast 2 (activity during step 2), and contrast 3 (step 2 – step 1). The detail design matrix and contrast used in GLM can be found in supplementary data. The GLM approach compared the covariance of each time point of the theoretical response to the actual response to create a *t*-test statistics for each channel. Unlike the classical ANOVA, GLM combined the experimental design and signal morphology to provide the best linear unbiased estimate of the hemodynamic response [26].

## 3 Results

**Figure 3:**
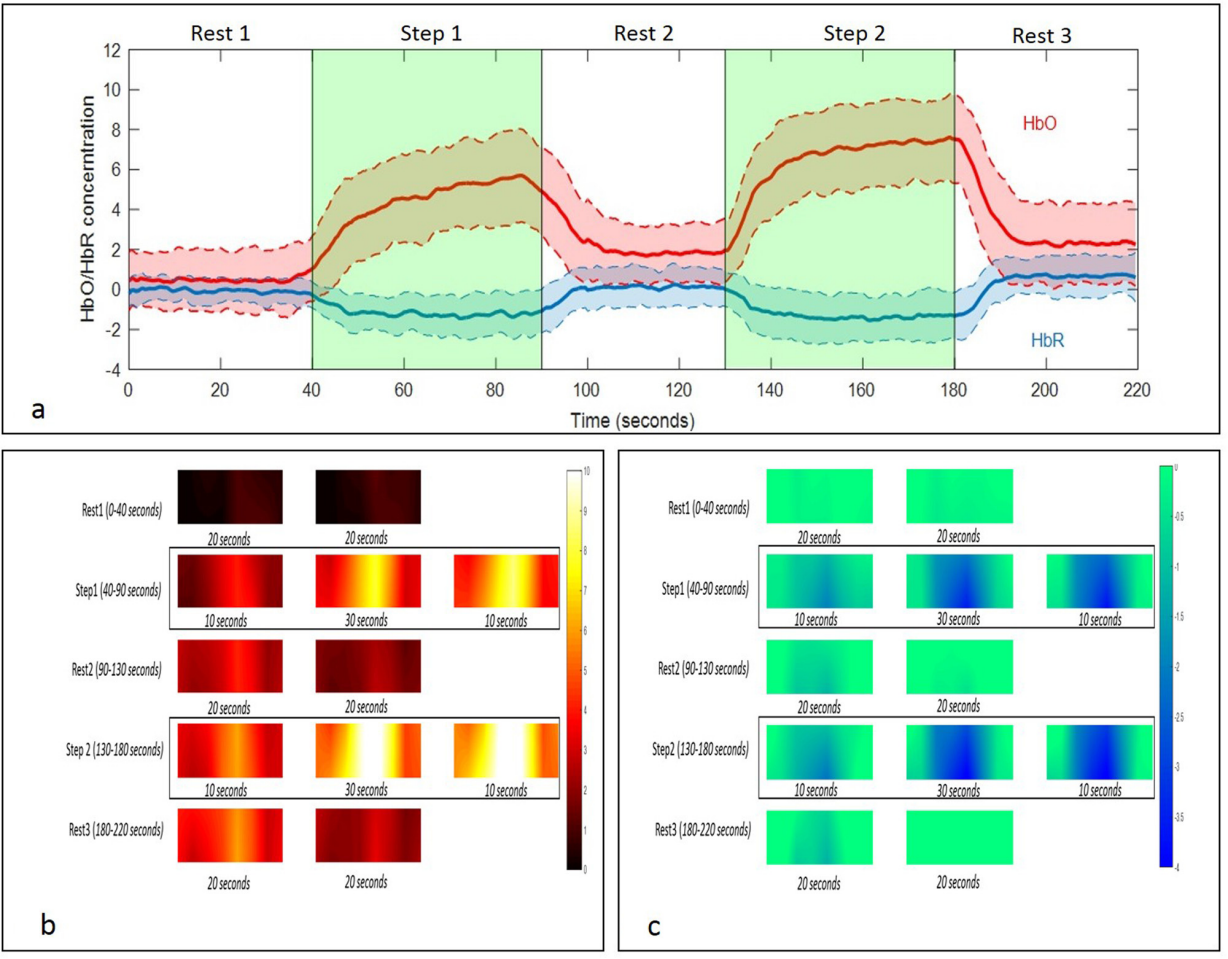
(a) The solid red and blue lines represent the grand average HbO and HbR concentrations across all optodes during the task, respectively. The shaded red and blue regions represent the standard deviation of HbO and HbR concentrations during the task, respectively. (b and c) Interpolated heat map of average HbO and HbR across all participants during the task.

On visual inspection, data from three participants were excluded because either the optodes had saturated signal or of the motion artifacts shifting the baseline. Thus, we were able to record prefrontal activity in 28 right-handed participants (8 females, 23.04 ± 2.60 years). Results of the data obtained from two left-handed individuals are mentioned separately in supplementary data. The participants were able to solve step 1 and step 2 with an accuracy of 99.29 ± 1.50% and 98.21 ± 2.64%, respectively, with no statistically significant difference between two accuracies (*p* = 0.1646, *t* = 1.429, df = 27). Figure 3a shows the grand average HbO and HbR levels across all optodes in all the participants. An increase in HbO levels (and decrease in HbR) was observed during step 1 and step 2. However, the HbO and HbR levels during the rest 2 period did not return to baseline values. Figure 3b and c shows the interpolated heat map of average HbO and HbR across all participants during the task. Furthermore, the step activity was divided into three parts, viz., initial 10 s (where HbO and HbR levels were either increasing or decreasing), middle 30 s (where HbO and HbR levels were relatively stable), and late 10 s (where HbO and HbR levels were either decreasing or increasing). Similarly, the rest period was divided into two parts, viz., initial 20 s and later 20 s. To evaluate the time issue, two-way ANOVA [task (step 1 and step 2) × 3 time (10, 30, and 10 s)] was performed (supplementary data).

**Figure 4:**
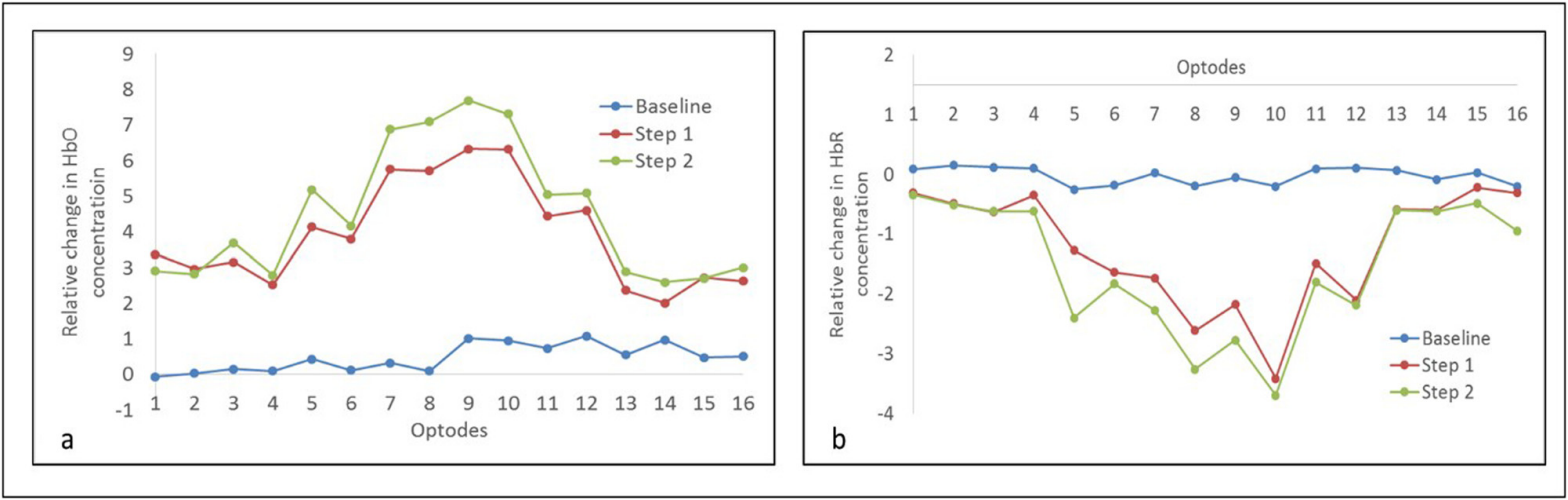
Grand average concentrations of HbO and HbR levels during baseline, step 1, and step 2 at 16 optodes locations for all participants. (a) and (b) represents comparison of relative HbO & HbR concentrations respectively.

We calculated the relative changes in HbO and HbR concentrations during step 1 and step 2 by subtracting the prestep rest activity. Average concentrations of HbO and HbR during step 1 and step 2 were calculated using a middle stable portion. Figure 4a and b depicts grand average concentrations of HbO and HbR levels during baseline, step 1, and step 2 at 16 optodes locations for all participants.

Two-way ANOVA was conducted that examined the effect of the task (baseline, step 1, and step 2) and 16 optodes locations on HbO and HbR. A statistically significant interaction was observed between task and optode locations on HbO, *F*(30, 1296) = 19.03, *p* < 0.0001, and HbR, *F*(30, 1296) = 41.91, *p* < 0.0001. *Post hoc* analysis showed a significant increase in relative HbO concentrations at all optodes during step 1 and step 2 when compared with baseline (*p* < 0.0001). In addition, a significant increase was observed in relative HbO concentrations at optodes 5, 7, 8, 9, and 10 during step 2 when compared with step 1 (Table 1). The HbR levels were significantly decreased at all optodes during step 1 and step 2 when compared with baseline (*p* < 0.0001). Moreover, HbR levels were significantly decreased at optode 4, 5, 6, 7, 8, 9, 10, 11, 15, and 16 during step 2 when compared with step 1 (Table 1).

**Table 1:** Adjusted *p* value at various optode locations after *post hoc* analysis (step 1 vs step 2 for HbO and HbR)

		Optode 1	Optode 2	Optode 3	Optode 4	Optode 5	Optode 6	Optode 7	Optode 8
Step 1 vs Step 2	HbO	0.2786	0.8849	0.1899	0.6629	**0.0019**	0.4658	**0.0007**	**<0.0001**
	HbR	0.6733	0.8703	0.9228	**<0.0001**	**<0.0001**	**<0.0001**	**<0.0001**	**<0.0001**

		Optode 9	Optode 10	Optode 11	Optode 12	Optode 13	Optode 14	Optode 15	Optode 16
Step 1 vs Step 2	HbO	**<0.0001**	**0.0037**	0.1219	0.2438	0.2074	0.135	0.9975	0.4406
	HbR	**<0.0001**	**<0.0001**	**<0.0001**	0.0958	0.9635	0.8144	**<0.0001**	**<0.0001**

Boldface indicates p values below 0.05, i.e. statistically significant.

**Figure 5:**
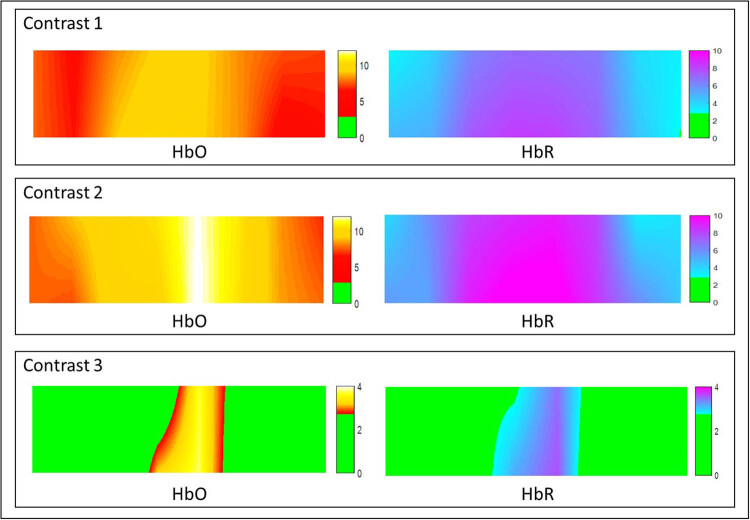
Statistically significant interpolated *t*-maps for HbO and HbR for three contrast models, viz., contrast 1 (step 1 – rest), contrast 2 (step 2 – rest), and contrast 3 (step 2 – step 1). *p* Value: 0.05 and its corresponding *z* threshold: 2.69.

Group analysis done using GLM in NIRS-SPM depicts the statistical significant *t*-maps for HbO and HbR for three contrast models, viz., contrast 1 (step 1), contrast 2 (step 2), and contrast 3 (step 2 – step 1; Figure 5). Significant activation was seen at all optode locations during step 1 and step 2 when compared with the rest (contrast 1 and contrast 2). In addition, A significant activation was seen during step 2 when compared with step 1 (contrast 3) in medial PFC regions corresponding to optodes 7, 8, 9, and 10.

## 4 Discussion

In this study, we were able to record the prefrontal cortical activity during Sudoku task in 28 right-handed participants and 2 left-handed participants. To our best knowledge, this is the first fNIRS study that tried to unravel the role of PFC during Sudoku task. To achieve this objective, the Sudoku task was uniquely divided into two steps that tried to separate the complex heuristic rule selection processes. Step 1 in our task eliminated the need for selecting a row or column rule. Thus, the PFC activity during step 1 represents simple processes such as searching empty boxes, remembering their location, finding the missing numbers, remembering the numbers, and placing the numbers while ignoring the stimulus coming from the nearby subgrids. Using the same grids to create a simple-level Sudoku (step 2) will now involve additional processes such as searching and applying the column and row rules. That is step 2 involves more complex heuristic rule selection processes. Thus, increased PFC activity during step 2 represents these additional processes. PFC is involved in many cognitive functions such as working memory, attention, and decision-making [27,28,29,30]. Thus, it is not surprising to find increased PFC activity during both steps of Sudoku task. Step 1 in our study is considered similar to the simple heuristic task and step 2 is similar to the complex heuristic task used by Wang et al. The researchers have shown that such complex tasks cost more time than simple tasks as cognitive processes involved in heuristic processing are complex [10]. A significant activation of left PFC was observed during the complex task when compared with simple task [8]. In this study, we saw that step 2 – step 1 contrast had medial regions activated. Indeed, these medial regions correspond to part of frontopolar cortex (FPC) which is proposed to be higher within the PFC hierarchy [31]. FPC is involved in behaviors that include complex decision-making [32], prospective memory [33], and rule-specific cognitive processing [34]. Also, activity in medial prefrontal regions is proposed to be decreased if trials become familiar [12]. Thus, increased activity in medial regions during step 2 – step 1 represents the processes involved and not due to familiarity. In other sense, it can be said that Sudoku as a leisure-time cognitive-stimulation activity helps in differentially activating the PFC regions. Thus, Sudoku can be used in cognitive remediation therapy especially for PFC. Indeed, sustained activity of brain regions during cognitive control-based paradigms enhance the performance of the trained task and also the benefits other domains [35]. Interestingly, the medial PFC is also important for coordinating the anticipatory responses to stress [36]. Thus, sustained activity and training of medial PFC can help the individual to mount better coordinated response to stress commonly seen in neuropsychiatric disorders such as depression. Thus, Sudoku-like social problem-solving therapy may be used for depression in elderly [37]. Sudoku involves logic and reasoning and it differentially activates PFC, and this puzzle may be used for schizophrenia patients where the patients fail to reason with reality. Future studies may be directed toward proving the beneficial effects of puzzles such as Sudoku in improving the PFC functionality in neuropsychiatric disorders.

Data acquisition and analysis methods used for understanding the task-related fNIRS signal are in their adolescent period when compared with fMRI analysis methods. In this study, we used both classical ANOVA and GLM methods to analyze the data to give convergent evidence of PFC activity during paper–pen block Sudoku task. However, the GLM approach supersedes the classical technique in reducing false negative and false positive results [38]. Nevertheless, newer fNIRS devices need to be used to rule out the effect of extra-cerebral hemodynamic changes, which can be achieved by adding short-channel regression techniques [39]. Moreover, future studies can be directed toward recording the complete cortex using multioptode fNIRS. Such recording will provide us with wider view of the networks involved during complex 9 × 9 Sudoku task processing. Future studies may also be directed toward the study of performance during Sudoku task. The advantage of such explorations will help us to device newer tasks in elderly or in patients requiring neurorehabilitation.

In this study, we compared the activity of PFC during Sudoku task versus at rest; and by dividing the puzzles into two steps, we explored the role of PFC during heuristic tasks. However, future studies may include a control condition to explain the heuristic effects. Moreover, the study was focused on PFC and thus future studies should test other areas to understand the frontoparietal and other brain region dynamics in Sudoku, as this will further establish the use of puzzles for cognitive rehabilitation. Moreover, the study must be replicated over larger population and in neuropsychiatric disorders to further establish its use.

To conclude, a significant involvement of PFC was observed during Sudoku task. Both the medial and lateral regions of PFC are activated when the participant is solving the Sudoku task. However, the medial regions of PFC play a differential role, especially when we consider the row and the column rules of Sudoku. Thus, Sudoku may be used for cognitive remediation training in neuropsychiatric disorders involving PFC.
